# Exploring the knowledge and attitudes of dentists towards immediate implant placements

**DOI:** 10.6026/973206300211207

**Published:** 2025-05-31

**Authors:** Divya Jyoti, Harmesh Sharma, Swarnim Singh, Manoj Kumar, Yogita Garg, Kalpana Rawat

**Affiliations:** 1 Department of Oral Health Sciences (Periodontics), Post Graduate Institute of Medical Education and Research, (PGIMER) satellite centre Sangrur, Chandigarh, Punjab; 2 Department of Periodontics, Government dental college, Patiala

**Keywords:** Dental, implant, success, attitude, knowledge

## Abstract

The integration of immediate implants into routine dental care varies considerably across different regions and practice settings. Therefore, it is of interest to describe theknowledge and attitudes of dentists towards immediate implant placements. A total of 225 eligible participants were required to have at least one year of clinical experience post-qualification. Participants were aged between 30-40 years (44%), followed by those aged 41-50 years (29%) and under 30 years (18). The majority of participants (72%) agreed or strongly agreed that immediate implants enhance patient satisfaction, while only 28% did not share this view. It is concluded that while the concept of immediate implant placement is widely recognized among dentists, substantial gaps remain in both knowledge and clinical confidence, particularly among general practitioners.

## Background:

Dental implantology represents one of the most transformative advancements in modern dentistry, providing a predictable and functional solution for replacing missing teeth [1]. The success rates and worldwide acceptance of implant therapy have increased due to innovative changes in surgical procedures and biomaterials development. Immediate implant placement stands out as a highly regarded technique because practitioners perform it by placing an implant right into an alveolar socket at the time of extraction for both clinical and psychosocial gains [2]. Improved patient satisfaction results from both bone tissue and soft tissue preservation and minimal surgical procedures, along with reduced treatment duration. The beneficial characteristics of immediate implant placement come with specific clinical challenges that practitioners need to address [3]. The clinical skills required for immediate implant placement must include achieving primary stability of fresh extraction sites and infection management while placing implants with accurate three-dimensional positioning and managing soft tissue contours [4]. The success rates of these procedures heavily depend on how skilled the professional is and how knowledgeable and experienced they are combined with their evidence-based approach [5]. The adoption of immediate implant treatments differs substantially depending on the region, together with the dental practice field where care is delivered [6]. The technique attracts opposing viewpoints from dentists since certain professionals consider it difficult and risky yet trained implantologists use immediate implants efficiently. Pluralistic professional attitudes towards clinical practice emerge mainly because students learn different subjects during university and receive different educational opportunities and teaching mentorship experiences in surgical fields [7]. Several studies from different countries showed that general dental practitioners possess significant levels of ignorance about immediate implant placement criteria which includes a traumatic extraction technique alongside socket assessment and bone quality evaluation and soft tissue control and appropriate patient selection [8]. The same importance level exists for the attitudinal component. Litmus tests about new techniques for dentists include both scientific proof alongside their assessment of patient needs and economic planning before considering risks [9]. Therefore, it is of interest to describe the knowledge and attitudes of dentists towards immediate implant placements.

## Methodology:

This cross-sectional descriptive study was conducted at Patiala and Ambala districts of Punjab and Haryana. A total of 225 dentists were included in the study. Participants were recruited using a convenience sampling method through dental networks, academic forums and professional groups on social media platforms. Eligible participants were required to have at least one year of clinical experience post-qualification. Dental students and interns were excluded to ensure that all responses reflected professional practice and decision-making based on real-world clinical exposure.

## Data collection:

The data were collected using a structured, self-administered questionnaire developed after an extensive review of the literature on dental implants and professional surveys in implantology. The questionnaire consisted of three major sections. The survey initiated by collecting vital demographic information consisting of practitioner age together with gender and years worked and specialty chosen and dental practice type. The assessment of knowledge regarding immediate implant placement took focus on clinical indications together with contraindications and healing protocols and technique sensitivity. The third section used a 5-point Likert scale to assess attitudes regarding implant placement by gauging practitioners' confidence level alongside their perceived advantages and disadvantages as well as their readiness for implementing immediate treatment in their clinical practice. Before finalization three implantology experts validated the content of the survey which received additional clarity and structure enhancement during testing with 20 professionals. The approved survey instrument was sent by electronic methods to participants. Survey participants could access the questionnaire only after agreeing to the informed consent document. The opening section specified that everyone took part in the study on a voluntary basis while offering protection through both privacy and confidentiality.

## Data analysis:

Data were analyzed using SPSS v25. Descriptive statistics, including means, standard deviations, frequencies and percentages, were used to summarize demographic data and general trends in knowledge and attitudes. Chi-square tests were used for categorical comparisons and independent samples t-tests were applied where continuous variables were involved. A significance level of p < 0.05 was used to determine statistical significance in all analyses.

## Results:

A total of 225 dentists participated in the study. The majority of participants in this study were aged between 30-40 years (44%), followed by those aged 41-50 years (29%) and under 30 years (18%). Most dentists had between 5-10 years of clinical experience (36%), while 30% had 1-5 years and 24% had 11-20 years. General practitioners made up 62.2% of the respondents, while specialists comprised 37.8%. Regarding sources of knowledge, continuing education courses were the most common (36%), followed by undergraduate training (20%), self-study (16%), workshops (15%) and postgraduate programs (13%) (Table 1). The overall mean knowledge score among all participants was 9.8 with a standard deviation of 2.6, indicating a moderate level of understanding. General practitioners had a lower average score of 8.9 ± 2.4, while specialists scored higher at 11.2 ± 2.1 (Table 2). The difference between the two groups was statistically significant with a p-value less than 0.01, suggesting that specialists had a better grasp of immediate implant placement concepts compared to general practitioners. The majority of participants (72%) (Table 3) agreed or strongly agreed that immediate implants enhance patient satisfaction, while only 28% did not share this view. However, confidence in performing the procedure was relatively low, with 57% indicating a lack of confidence. A considerable proportion (38%) admitted fear of complications, while 62% did not consider it a concern. Just over half (52%) correctly identified the importance of an infection-free site before implant placement, while 41% recognized the need for achieving primary stability. A larger portion (60%) was aware that immediate implants reduce overall treatment time. However, only 28% knew the correct minimum torque requirement of 30 Ncm, indicating a notable gap in technical understanding (Table 4). Only 32% of general dentists reported confidence in performing the procedure, compared to 61% of specialists (p < 0.01). Similarly, just 18% of general dentists had previously placed an immediate implant, while over half of the specialists (56%) had done so (p < 0.001) (Table 5). Additionally, 81% of specialists had received continuing dental education (CDE) on implants, significantly more than the 49% of general dentists (p < 0.001), highlighting the impact of advanced training on clinical readiness. A majority (57%) responded "Yes," indicating a strong interest in adopting the procedure. Meanwhile, 19% expressed unwillingness and 24% were unsure (Figure 1 see PDF).

## Discussion:

The present study aimed to evaluate the knowledge and attitudes of dentists regarding immediate implant placement, a technique gaining momentum for its potential to reduce treatment time and improve patient satisfaction. The participants exhibited an average knowledge level as evidenced by the moderate scores (mean = 9.8 ± 2.6) yet specialists scored better than general dentists. The study results match previous academic evidence showing that specialized clinical training leads dental professionals to achieve better outcomes in complex procedures such as implantology. The low rate of correct responses from general practitioners regarding infection control and torque requirements supports the need for better education programs at both baseline and continuing education stages [10, 11]. Many practitioners recognized the benefits of immediate implant procedures yet only 43% felt capable to carry out this technique without assistance. The survey results showed that 65% of clinicians wanted additional training because they showed an interest in newer techniques as long as appropriate educational resources and support are given to them [12]. The reluctance stems from a general lack of commitment towards surgical practices lacking proper framework support or skilled instruction particularly in private medicine practices which exhibit strong risk avoidance tendencies [13]. Clinical decisions making demonstrates various aspects because cost analysis and patients who rejected procedures or displayed anxiety were recorded by healthcare providers [14]. The evaluation of general practitioner experience compared to specialist experience demonstrated important differences regarding clinical work exposure and professional perspectives. Data revealed that specialists showed a considerably higher rate of experience with immediate implant placements and attendance in implant-specific training programs [15]. It highlights that while most interns have a generally positive attitude towards dental implantology, there are significant gaps in their practical experience and depth of theoretical knowledge. The findings suggest the need for enhanced implant education in dental curricula and more clinical exposure to ensure that future dentists are well-prepared to offer implant treatments confidently and competently [16]. Official training programs together with practical surgical encounters create variations which determine how prepared clinicians become for their professional duties. Surprisingly a substantial 24% of all respondents expressed doubt about their future intentions to perform immediate implants despite 57% indicating their willingness. The research supports global patterns which demonstrate dental implantology needs continued professional growth for dental professionals. General medical practices in countries that successfully integrated implantology treatments feature solid continuing education systems together with formal clinical mentorship systems.

## Conclusion:

The concept of immediate implant placement is widely recognized among dentists. However, substantial gaps remain in both knowledge and clinical confidence, particularly among general practitioners. Majority of participants were aware of the general benefits of immediate implant procedures. Nonetheless, a detailed understanding of critical clinical factors, such as torque requirements, infection control and primary stability is needed.

## Figures and Tables

**Table 1:** Demographics of participants

**Characteristic**	**N (%)**
Age <30 years	41 (18%)
Age 30-40 years	99 (44%)
Age 41-50 years	65 (29%)
Age >50 years	20 (9%)
Experience 1-5 years	68 (30%)
Experience 5-10 years	81 (36%)
Experience 11-20 years	54 (24%)
Experience >20 years	22 (10%)
General Practitioners	140 (62.2%)
Specialists	85 (37.8%)
Source of Knowledge	
Undergraduate Training	45 (20%)
Postgraduate Program	30 (13%)
Continuing Education Courses	81 (36%)
Self-Study (Books/Journals)	36 (16%)
Workshops/Hands-on Training	33 (15%)

**Table 2:** Knowledge scores by group

**Group**	**Mean ± SD**	**p-value**
All Participants	9.8 ± 2.6	
General Practitioners	8.9 ± 2.4	< 0.01
Specialists	11.2 ± 2.1	

**Table 3:** Attitudes towards immediate implant placement

**Statement**	**Agree (n, %)**	**Strongly Agree (n, %)**	**Not Agree (n, %)**
Immediate implants enhance patient satisfaction	102 (45%)	60 (27%)	63 (28%)
Confident performing immediate implant procedures	61 (27%)	36 (16%)	128 (57%)
Reluctant due to fear of complications	51 (23%)	35 (15%)	139 (62%)
Interested in further training or workshops	89 (40%)	57 (25%)	79 (35%)

**Table 4:** Knowledge assessment results

**Knowledge Item**	**Correct Responses (n, %)**
Correctly identified need for infection-free site	117 (52%)
Recognized need for primary stability	92 (41%)
Aware of reduced treatment time benefit	135 (60%)
Knew minimum torque requirement (≥30 Ncm)	63 (28%)

**Table 5:** Attitude comparison between general dentists and specialists

**Attitude Statement**	**General Dentists (n, %)**	**Specialists (n, %)**	**p-value**
Confident performing immediate implant procedure	45 (32%)	52 (61%)	< 0.01
Previously placed an immediate implant	25 (18%)	48 (56%)	< 0.001
Received implant training (CDE)	69 (49%)	69 (81%)	< 0.001
